# The Sequelae of Metallosis Resulting in Skin Pigmentation and Tattooing: A Case Presentation and Literature Review

**DOI:** 10.1055/s-0036-1596060

**Published:** 2016-12-14

**Authors:** Stephen Thomas, Conor Gouk, Narlaka Jayasakeera, Michael Freeman

**Affiliations:** 1 Division of Dermatology, Department of Orthopaedics, Gold Coast University Hospital, Southport, Queensland, Australia

**Keywords:** metallosis, metallosis treatment

## Abstract

With advancing technologies in orthopedics and increasing demands of the population for orthopedic interventions, younger patients are now receiving joint replacements. One of the potential risks of joint replacement is metallosis, or the local and systemic release of metal ions. Metallosis is caused by the release of metallic debris, secondary to hardware failure. The phenomenon is most commonly associated with failed metal-on-metal hip prostheses and is characterized locally by heavy staining of surrounding soft tissue, metallic synovitis, joint effusion, and gradual loosening of the prosthesis. Additionally, metallic debris can also lead to periarticular superficial skin manifestations. The release of metal ions has further been known to lead to systemic upsets including neurologic deficit (declining vision, hearing, or cognition; headaches), cardiac failure, and hypothyroidism. As the number of patients seeking major orthopedic interventions grows, the incidence of metallosis-related skin tattooing will also increase. The structural components of a failed joint replacement can be revised (improving patients' pain and functioning). However, any skin tattooing secondary to metallosis presents the treating dermatologist with clinical challenge, due to lack of research regarding treatment of this condition. Our aim is to review the published literature on metallosis, including the pathophysiology. After assessing publications on the treatment of traumatic and cosmetic tattooing, we hope to stimulate further research regarding treatment. This article should also serve to remind orthopedic surgeons that with increasing patient concern regarding cosmesis, a multispecialty approach including referral to a dermatologist is valuable.

The advent of joint replacement surgery has allowed patients with joint pathologies to return to being pain-free and functional in the effected site to a level not previously possible. As the number of patients seeking orthopedic intervention grows, joint arthroplasty is becoming more common.

A potential risk of joint replacement surgery is metallosis, caused by the release of metallic debris secondary to hardware failure. Metallosis can be either local or systemic and has been observed at an estimated incidence of up to 5% in metal joint replacement patients. Metallosis may present locally with pain, instability, and metallic debris staining the local tissue. Systemically, metallosis has been linked to neurologic complications (visual, hearing, and cognitive deficits), cardiac failure, and hypothyroidism.

Importantly, metallic debris can also have superficial manifestations of periarticular skin tattooing, due to an adverse local tissue reaction. Although the structural component of failed joint arthroplasty can be revised to improve pain, stability, and functioning, any skin tattooing secondary to metallosis presents the treating physician with clinical challenge.

Two factors determine the survivorship of an implant: metallurgy and implant design. Both factors are interrelated and help to determine biological responses to the implantation of a prosthesis. 1 Metallurgy refers to the physical and chemical behavior of metallic elements. The most common materials used for orthopedic prostheses include stainless steel, chromium-cobalt alloy, and titanium alloy, each of which may contain nickel, cobalt, chromium, titanium, molybdenum, aluminum, iron, manganese, copper, tungsten, or vanadium. 2 Implant design and loading vectors associated with that design also contribute to implant survivorship.

This study presents a review of pertinent medical literature on the subject of metallosis.

## Case Study

**Fig. 1:**
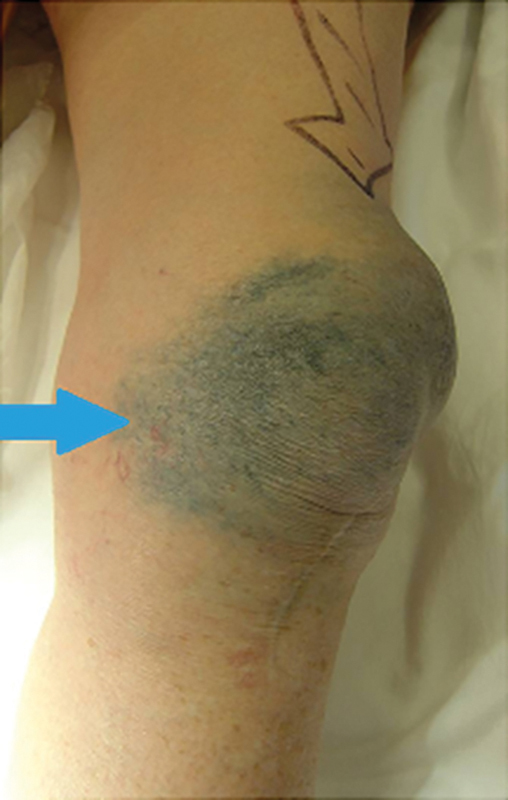
Skin discoloration and joint effusion (arrow) after knee arthroplasty failure, prior to revision surgery.

Jayasekera et al reported a 74-year-old woman who presented to her general practitioner after experiencing 2 years of worsening metallic skin tattooing, pain, and swelling to her left knee ( Fig. 1 ). 3 Six years earlier, the patient had left total knee replacement, with a metal-backed implant to resurface her patella, and an anterior knee midline scar.

The patient underwent joint revision, where periarticular stained soft tissues of the knee were debrided (primarily joint capsule/synovium), and the patella component of the prosthesis was replaced with an alternative. The patient remained pain-free with good function at 5-year follow-up, but had ongoing concern about cosmesis of her persistent skin tattooing, which remained untreated.

## Materials and Methods

A comprehensive literature search was performed, following the PRISMA 27-point checklist, and taking into consideration the guidance offered by the Cochrane Collaboration. We completed a systematic review of PubMed, Embase, MEDLINE, and the Cochrane Library, from 1975 to June 2015. We used the search terms “metallosis,” “treatment of metallosis,” “traumatic tattooing,” “tattoo removal,” and “tattoo treatment,” which returned 1,806 articles. Screening of the title and abstract was performed by two of the authors (S.T. and C.G.), which helped identify the relevant articles. From these, an additional search was performed through reference lists of the retrieved articles. Our search yielded 10 articles for our review of the treatment of skin discoloration following metallosis.

## Results

Twelve studies matched the search criteria and were included in our review. Four studies dealt with clinical cases of metallosis. Three different studies dealt with the histopathology of the condition, and the remaining five studies were related to assessing treatment of skin tattooing.

### Metallosis Post–Joint Arthroplasty

Babis et al reported a 70-year-old woman with a right total hip replacement, with a history of developmental dysplasia of the hip from childhood, who presented with severe pain and periarticular skin metallic staining. 4 On joint revision, the porous tantalum acetabular component of the hip replacement had failed. Serum levels of tantalum had increased 2,000 fold and subsequently dropped by 25% 6 months after re-revision (tantalum implants were reused).

Munro-Ashman and Miller patch-tested 35 patients who suffered metal-to-metal prosthesis failures. 5 A total of 35 patients in this unsatisfactory group were patch-tested; 16 were positive to metals, 13 to cobalt, 4 to nickel, and 2 to chromate. Only two patients showed any skin lesions—one a localized dermatitis around the knee joint from nickel sensitivity, and one with widespread scattered circular erythematous lesions suggestive of a generalized allergic vasculitis from cobalt exposure.

The authors recognized that roughly half of arthroplasty failures may be related to metal sensitivities (particularly cobalt) and suggested taking a careful history for metal sensitivity and patch-testing high-risk patients with the metals. Titanium 318 was suggested as a satisfactory substitute for cobalt chrome alloy if reactions were encountered or anticipated.

Bradberry et al systematically reviewed toxicities attributed to metals released from hip implants, surmising that high circulating concentrations of cobalt from failed hip replacements could cause neurologic damage, hypothyroidism, and/or cardiomyopathy, which may fail to reverse after joint revision or removal. 6

Metallosis can lead to osteolysis and subsequent implant failure, which may be seen in noninvasive plain radiography. An often nonspecific radiographic sign observed is periprosthetic osteolysis; more specific signs that have been reported are the “bubble,” “cloud,” and “metal-line” signs. 7 8 Serologic testing for metallic ions reveals elevated levels. A normal serum cobalt level is around 0.19 μg/L, with levels greater than 5 μg/L posing risk of neurologic and cardiac abnormalities. 9

### Histopathology of Metallosis

Asahina et al reported a 59-year-old woman with a background of rheumatoid arthritis who experienced metallosis tracking to the distal end of the forearm, several centimeters beyond the prosthesis location, several months after elbow arthroplasty. 10 The joint remained fully functional; she was pain-free and had no lymphadenopathy. Histopathology showed fine brown-black particles dispersed in the dermis, phagocytosed by macrophages, or else accumulating around eccrine glands and capillaries, indicating an adverse reaction to metal debris. X-ray analysis of the tissue showed high levels of iron, nickel, and chromium.

The authors theorized that problematic metallic particles in metallosis follow a random diffusion throughout soft tissue. Macrophages then attempt to phagocytose these foreign bodies following failure of the mechanism to remove them from dermal tissue. It was also suggested that the origin of the metallic staining was secondary to precipitation of the metallic particles from the instruments used during the arthroplasty procedure.

Matziolis et al noted a case of metallosis following a total hip replacement, where histopathology showed numerous histiocytes and foreign body giant cells containing fine metal (cobalt-chromium) particles and moderately dense lymphoplasmacytic infiltrates. 11 The metal particles appear as extracellular metal deposits or as debris in the cytoplasm of histiocytes and foreign body giant cells (hematoxylin and eosin staining, ×400 focus).

Akimoto et al noted an 80-year-old woman, who presented with metallosis mimicking a malignant skin tumor 6 years after right total hip arthroplasty. 12 Histology showed diffuse granulomatous inflammation, large lymphatic spaces, and fine brown-black granules within the histiocytes (likely originating from the titanium acetabular cup and screws). The authors considered that in this case, the granulomatous tumorlike reaction was possibly a more severe immunologic reaction to the inflammatory metallic debris products.

### Treatment of Skin Tattooing with Laser Therapies

No articles were located assessing treatment of metallosis. However, articles assessing treatment of other forms of cosmetic and traumatic skin tattooing were found.

Apfelberg et al compared treating decorative tattoos using argon laser with using CO _2_ laser and mechanical debridement. 13 He found similar results, complication rates, and consequential histologic studies between the two groups.

Bernstein et al treated a 60-year-old woman with a traumatic tattoo from a residual deep dermal white braided suture (Polysorb, Covidien), 14 which presented as a green discoloration along the length of an elliptical surgical scar from a basal cell carcinoma excision on the right upper lip. A 3-mm punch biopsy showed green, vertically oriented pigment within the reticular and papillary dermis, without any histologic evidence of a foreign body reaction. The discoloration was treated with Q-switched ruby laser (694 nm for 28 nanoseconds) over two treatments and cleared.

Troilius assessed the use of the Q-switched neodymium:yttrium aluminum garnet (Nd:YAG) 1,064-nm laser for treatment of 12 patients with traumatic tattoos after accidents. 15 It was found cosmetically successful for gravel, mascara, or high explosives in two to five treatments. Asphalt, amalgam, and metal took 6 to 11 treatments for successful treatment.

Gorouhi et al looked at the treatment of traumatic tattoos with Q-switched Nd:YAG 1,064 nm with spot size 4 mm, fluence of 7.96 J/cm ^2^ . 16 One 54-year-old man with sand and asphalt tattooing on his face following a bomb explosion 15 years prior received the laser treatment. After a test area was performed, one treatment gave excellent outcomes. No pigmentary or textual changes were noted. Gorouhi et al theorized that these lasers may optimize tattoo removal either by increased phagocytosis or through transepidermal elimination. 16 It was noted that multiple treatments with Q-switched lasers were required for deeper traumatic tattoos.

Sunde et al looked further at the treatment of established traumatic tattoos. 17 The authors developed an animal model for traumatic tattoos where two levels of wounds were made (shallow and deep). Each group consisted of five guinea pigs with one consistent level of wounding. Four treatment methods were applied, including carbon dioxide laser, argon laser, overgrafting, and dermabrasion. The results were evaluated by trained observers on a gross basis. Although no statistically significant differences were found within these small groups, clinical experience in a small group of patients suggested that carbon dioxide laser may prove to be useful in the delayed treatment of traumatic tattoos. Eight patients were treated over a 4-year period. Satisfactory total or subtotal foreign body removal of various agents (road tar, cement, cooper particles) was largely achieved.

## Discussion

Metallosis is an uncommonly encountered complication of joint arthroplasty. The orthopedic surgeon can provide initial management, including investigations for systemic involvement, appropriate joint revision and tissue debridement, and referral to necessary specialties. The surgeon should consider referral to the dermatologist for assistance in managing the patient's cosmetic expectations after optimal surgical management has failed.

The orthopedic surgeon should also be cautious to recognize patients at increased risk of sensitivity-based prosthetic failure and should consider use of the appropriate compounds when initially assessing the patient.

Histologically, metallosis involves metallic particles migrating from the deeper periarticular tissues superficially to the dermis, with varying immunologic reactions. There is little in the current literature to recommend an optimal laser treatment; current research looks at treatment of tattoo particles introduced superficially and less likely to be concentrated in deeper tissues (as in metallosis).

Adequate treatments with the fractionated CO _2_ ablative laser, or the Q-switched Nd:YAG 1,064-nm laser, capable of either allowing the particles to be released from the dermis (transepidermal elimination) or breaking up the particles at least in the dermis, are likely to be successful. Although particles deeper than the dermis would fail to be treated with laser, it is our view that the dermal particles likely contribute greater to the appearance, increasing the probability of laser treatment to be effective.

Because no current research into laser or other treatment of metallosis has been conducted, clinical trials would be of benefit. This would help guide the physician toward optimal therapy when addressing this condition.

## Conclusion

We conclude that for successful management of skin metallosis, the orthopedic surgeon must revise the arthroplasty, addressing all relevant factors, and seek referral to the dermatologist as appropriate. Although pain and mechanical symptoms can be addressed with joint revision, cosmesis is also important when considering the patient's best interests and long-term outcomes. Although dermatologic literature regarding metallosis treatment was not found, deduction from treating similar conditions can guide the physician toward optimal management. Possible limitations to our review may include the restrictions of the MEDLINE-based search, the lack of statistical analysis, and the small number of studies considered. Given the relatively few studies assessed, it is our opinion that a statistical analysis would not influence the findings of our review.

Although other dermatologic treatment options exist (including dermabrasion, local excision, and overgrafting), these modalities are likely suboptimal and require a further evidence basis before being utilized to manage metallosis. We hope this review stimulates further research, guiding future dermatologic investigation and trial of treatment.
